# The ECHO study: compassion-focused therapy for young voice hearers and their caregivers. A pilot study

**DOI:** 10.3389/fpsyt.2026.1827986

**Published:** 2026-07-10

**Authors:** Maja Kargaard Musaeus, Charles Heriot-Maitland, Vibeke Fuglsang Bliksted

**Affiliations:** 1 Research Unit of Psychiatry Odense-Svendborg, Department of Clinical Research, University of Southern Denmark, Odense, Denmark; 2 Balanced Minds, Edinburgh, United Kingdom

**Keywords:** adolescent, CFT, schizophrenia, voice hearing, youth, caregiver, psychosis

## Abstract

**Background:**

Voice hearing can occur in healthy people without a trigger, be caused by a traumatic event, or occur as part of a psychiatric disorder. There are still many gaps in our understanding of voice hearing, and research in this field can be challenging, maybe because it is associated with shame and stigma. Those who experience distress by voice hearing could benefit from a treatment, aimed directly at that. The ECHO manualized treatment is based on the principles of compassion-focused therapy (CFT).

**Methods:**

Treatment will take place on several different psychiatric hospitals in the Region of Southern Denmark. Treatment consists of a 10-session, manualized CFT for voice hearing. Caregivers participate in half of the sessions, and a recording of the voice content is made, which the caregivers are encouraged to listen to. The study is designed as a prospective single-arm pre–post intervention study using a mirror-image approach, in which participants serve as their own controls over time. The intervention group consists of (*N* = 60) voice-hearing adolescents aged 13–18 years. Additionally, a clinical comparison group (who also receive the intervention) of (*N* = 20) individuals aged 18–20 years with first-episode schizophrenia and voice hearing is included for descriptive and exploratory analyses. Data are collected 3 months prior to CFT treatment, immediately before the start of treatment, immediately after treatment is completed, and at 1 month follow-up. Data will be centered around social cognition, perceived social safeness, social connectedness, general well being, auditory hallucinations and compassion.

**Discussion:**

The ECHO study addresses the wellbeing and thriving of an overlooked group of young people. The results of this study will provide information about who the voice hearers are, what challenges they face, and how they perceive them. The results will also provide information about the feasibility and efficacy of the treatment manual and, thus, its potential further implementability. Caregivers participate in half of the sessions, to facilitate a common language about mental states and voice hearing. To support their understanding, a recording of the voice content is made and the caregivers are encouraged to listen to it. We suggest that when caregivers understand the young voice hearers’ experiences, they can adjust their expectations and approach accordingly. We also suggest that by sharing the voice content, the young voice hearer will feel less isolated.

**Study protocol registration:**

https://clinicaltrials.gov/study/NCT07314515, identifier NCT07314515.

## Background

There can be various reasons why you experience voice hearing. For some people, it is a part of their day-to-day life, with no distress associated with it (1), while others only experience it when emotionally burdened due to difficult life experiences. Yet, others experience it as a symptom and meet the criteria for an actual psychiatric disorder. Voice hearing (hearing voices that other people cannot hear, without there being a corresponding external auditory stimuli) is considered an auditory hallucination (AH). AHs can take many different forms; however, in this study, we focus on the ones that take a spoken and understandable form and refer to this phenomenon as voice hearing. This form of AHs can also be considered hallucinated social communication (2).

Voice hearing can occur in a wide range of different psychiatric diagnoses such as depression, mania, autism, post-traumatic stress, personality disorders, and primary psychotic disorders, including schizophrenia (3, 4). In addition, people with major cognitive challenges (low intelligence) also have a low psychosis threshold (5). It is difficult to know exactly how many young people hear voices at some point in their youth, as there are still a lot of stigmas associated with it. It has been estimated that between 5% and 7.5% of all 13- to 18-year-olds experience some degree of voice hearing (6). Despite greater openness about mental illness, voice hearing continues to be associated with great shame, which can exacerbate the patient’s suffering and reduce their chances of recovery (7). A recent review found that AH was the most frequent symptom (81.9%) in schizophrenia spectrum psychosis in children and adolescents, and these young patients had longer duration of untreated psychosis than patients with adult-onset schizophrenia (8).

Compassion-focused therapy (CFT) refers to the underpinning theory and process of applying a compassion model to psychotherapy. It is an approach that draws from evolutionary, social, developmental, and Buddhist psychology as well as neuroscience. One of its key concerns is to use compassionate mind training to help people develop and work with experiences of inner warmth, safeness, and soothing, via compassion and self-compassion (9). In CFT, three systems are described: the threat system, the drive system and the soothing system (9). In CFT, the aim is to find a balance between the three systems, while at the same time trying to develop a compassionate mind that is sensitive to your own suffering, and motivated to prevent and alleviate this suffering. CFT not only aims to understand mental health diagnoses but also seeks to explain psychiatric symptoms as psychological responses to life experiences. In this context, voice hearing in adolescents can be understood as a reaction to stressful or traumatic experiences (10, 11).

In working with voice hearers, CFT aims to help them to first notice their threat-based system when it arises and, secondly, understand its function within the context of their lives, and how to activate the soothing system through compassion. CFT aims to help people develop their capacity for affiliative relating (with self, voices, and other people; 12).

The treatment in the current research project is a modified version of a manualized CFT treatment targeted at adults with voice hearing developed by Charles Heriot-Maitland (13). Over the 10 sessions, the young voice hearer and their caregiver are introduced to CFT; this includes the “tricky brain” and the three systems already mentioned. The “tricky brain” is the evolution-shaped mind that often defaults to threat, self-criticism, and survival responses, even though those responses can cause emotional suffering (9). Patients are also introduced to exercises that are intended to activate the soothing system. These are guided soothing breathing practice and imagined scenarios such as an ideal compassionate other and a safe space. Where the young person is invited to use their imagination to activate their soothing system. The CFT has so far not tested young voice hearers. However, studies have shown that young people experience greater autonomy in relation to the voices if you study the intentions of the voices together with the young person (14). Caregivers play an important role in relation to how a young voice hearer experience and cope with their symptoms, but in particular, mothers feel that they are not sufficiently involved in their child’s psychiatric treatment, when it comes to voice hearing (14). Studies have also shown that clinicians and parents may lack the courage and knowledge of how to talk to the young person about the content of the voices (15). Finally, research indicates that there may be an association between voice-related stress and social relationships (16, 17).

## Purpose and relevance

In the current research project, a manualized CFT manual is being tested, which, at the same time, also involves the patient’s primary caregivers in the treatment. In addition to traditional CFT, the treatment is expanded with an intervention where an audio file is recorded with content corresponding to the adolescent’s voice hearing. The primary caregivers are invited to listen to the audio file while participating in a therapy session. This will help improve the caregivers’ understanding of the young person’s experiences and challenges. Together with the therapist, they will attempt to understand which system the voices are talking from, and what they are trying to motivate the young voice hearer to do. The treatment is transdiagnostic and consists of 10 sessions, which is considered a realistic duration in relation to clinical resources. The original CFT manual (compassionforvoices.com) was slightly modified to ensure that the instructions and examples could be understood by young voice hearers. Duration and number of sessions have been approved by the original author, Charles Heriot Maitland. Furthermore, we added text to involve the primary caregivers in the psychoeducational part of the treatment. Finally, we added tips and tricks for the clinicians to ensure that they would be well equipped using the manualized treatment.

## Objectives

This is a pilot study, where we will investigate the clinical feasibility and efficacy of a 10-session CFT treatment, where primary caregivers are closely involved in the treatment of young voice hearers.

We will also collect data, e.g., achieve knowledge of which specific treatment outcomes are targeted with this CFT manualized treatment. Finally, we collect evaluations about the treatment from young voice hearers and their primary caregivers.

By comparing young voice hearers to patients with first-episode schizophrenia (FES), we hope to be able to identify potential subgroups of young voice hearers that might have a larger need for further preventive psychiatric treatment. Furthermore, we will investigate the association between voice-related distress and perceived social support.

## Hypotheses

Baseline data (3 months before CFT treatment):

1. We expect to find an association between greater severity of psychotic symptoms and less voluntary control over voice hearing.
2. We expect to find an association where patients with the most severe psychotic symptoms have more social cognitive deficits and less social connectedness, social pleasure, and quality of life.

Effect of the CFT treatment (right before treatment, when treatment is finished, and at 1 month follow-up):

3. CFT treatment will improve patients’ feeling of voluntary control over voice hearing.
4. CFT treatment will improve social connectedness to primary caregivers, e.g., perceived social support and social pleasure.
5. CFT treatment will minimize amount and severity of voice hearing.

Comparison of young voice hearers to patients with FES.

6. We expect that a subgroup of the young voice hearers with the most severe voice hearing will resemble the patients with FES in terms of social cognitive deficits, voluntary voice hearing control, social connectedness, and social pleasure compared to the less affected young voice hearers.

## Methods, materials, and measures

We include 60 patients with voice hearing aged 13–18 years and 20 patients with newly diagnosed schizophrenia aged 18–21 years. The pilot study will have one arm, but two subgroups of participants. We use a mirror design where the participants are their own control person. The reasoning behind this is that the participants are recruited from different psychiatric units, and none of them offer a treatment specifically for voice hearing but is more centered on a psychiatric diagnosis. Because of this, treatment as usual can be very varied. By using a mirror image study design, we can compare the standard treatment (treatment as usual) that the patient receives before entering the project to the effects of the manualized CFT treatment. Data are collected 3 months prior to CFT treatment, immediately before the start of treatment, immediately after treatment is completed, and at 1 month of follow-up. This pilot study adhered to CONSORT guidelines for pilot and feasibility trials. The next future step will be a formal RCT study based on the results from the current project.

Approvals to get access to patients have been granted from OPUS, Psychiatric Department Odense and the management from Child and Adolescent Psychiatry, Region of Southern Denmark.

A detailed CFT treatment manual has been prepared, which contains thorough descriptions of the content of the 10 therapy sessions. The parents participate in five of these sessions. The original idea for the research project comes from specialist psychologist Maja Musaeus and her experiences from working with young patients with psychosis in Child and Adolescent Psychiatry in Esbjerg. The treatment manual has been prepared by Maja Musaeus in collaboration with Dr. Charles Heriot-Maitland and Prof. Vibeke Bliksted. The manual has been tested to help estimate the time needed to complete a session and to test if the language seemed fitting for the age group. Sessions took, on average, 40 min, and the language seemed fitting. Feedback from one of the patients was that “she wasn’t tormented by the voices anymore, because their content had stopped being demeaning”.

There are no known side effects of CFT.

The clinical therapists receive a thorough 2-day training in the method from Charles Heriot-Maitland, Maja Musaeus and Vibeke Bliksted.

Maja Musaeus and Charles Heriot-Maitland supervise the CFT treatment at monthly meetings throughout the data collection period. This is done to ensure treatment fidelity. Student assistants collect research data. Vibeke Bliksted and Maja Musaeus are responsible for training and supervising student research assistants.

All questionnaires are entered directly into the database program RedCap (18, 19). The data collection is expected to be completed in 1–2 h (depending on the need for breaks along the way). All questionnaires have been tested on a similar aged population, for time estimates and feasibility.

Inclusion criteria were as follows: 13- to 18-year-old patients with voice hearing, 18- to 21-year-old patients with First episode schizophrenia (FES) and voice hearing, an estimated IQ above 70 based on school achievements and background information, no drug use while attending therapy sessions, and a primary caregiver who can participate in five of the CFT sessions.

Exclusion criteria were as follows: Brain injury or neurological disorder, e.g., severe epilepsy; unable to speak or understand Danish (as the need for interpreter assistance will make it difficult to carry out manualized treatment); and visual and hearing impairment (which will make it difficult to carry out the manualized treatment).

## Measures

### The young person

Social cognition is tested with TASIT 2A DK (The Awareness of Social Inference Test – Social Inference Minimal) (20). Additionally, questions are asked about how well you feel your parents understand you (e.g., do they know what motivates you and what scares you)? (Likert scale 1–10). Questions about daily stress, sleep, violence and abuse, wellbeing and quality of life, and interaction with family and friends are taken from Hvordan har du det? -- Central Denmark Region’s large-scale health profile survey, conducted every four years, which assesses the health, morbidity, and wellbeing of the general population (21). Quality of life is measured with WHO-5.

AHs are measured with three different questionnaires. Interpretation and relationship with the voices are measured with BAVQ-R (Beliefs about Voices Questionnaire – Revised) (22). Severity and phenomenological characteristics of voice hearing are measured with PSYRATS AH (Psychotic Symptoms Rating Scale Auditory Hallucinations) (23). Perceived ability to control or influence the voices is measured with The Yale Control Over Perceptual Experiences (COPE) (24).

Subjective experience of feeling safe, accepted, and soothed in social relationships is measured with SSPS (Social Safeness and Pleasure Scale) (25). Compassion toward self is measured with SCS-SF (Self-Compassion Scale Short Form) (26, 27).

Subjective sense of interpersonal closeness and belonging is measured with The Social Connectedness Scale Revised (28, 29). Perceived availability and adequacy of social support is measured with MSPSS (The Multidimensional Scale of Perceived Social Support) (30).

### The caregivers

The caregivers are asked to confirm the young person’s level of education, the civil status of the parents, the young person’s residence, family history of mental illness, and the diagnosis and medication of the young person. They are also asked how well they feel they understand the young person (what motivates them and what scares them?) (Likert scale 1–10).

## Feasibility

Patients aged 18–21 years with schizophrenia will be recruited from OPUS, the Clinic for Newly Diagnosed Schizophrenia, at the Local Psychiatry in Odense. Patients aged 13–17 years experiencing AHs will be recruited from the Child and Adolescent Psychiatry in the Region of Southern Denmark. Based on data from the Central visitation of the Child and Adolescent Psychiatry in the Region of Southern Denmark, we expect 190 new young patients with psychotic symptoms annually. There are four Child and Adolescent Psychiatric departments, and we expect to train two clinicians at each site. Enrolling 60 patients over a 2-year period will amount to seven to eight from each site per year, which has been approved by the head of the Child and Adolescent Psychiatry in the Region of Southern Denmark. The feasibility of the study will be assessed through the following question: Is the treatment acceptable for young people and their caregivers? Assessment will be based on the number of patients who want to participate and the number of patients who complete the intervention. The study aims to enroll participants continuously until 50 participants has completed the intervention or until recruitment is terminated due to time constraints. It will be considered optimal, should more than *n* = 50 participants complete the intervention (31).

### Ethics

The study had been approved by the Region of Southern Denmark Committees on Health Research Ethics (Projekt-ID S-20240085 Acadre nr: 24/49956) and the legal data Fortegnelse (Journal nr.: 24/52212). The project complied with the Declaration of Helsinki of 1975, as revised in 2008. All personal information and research data will be kept according to the GDPR regulations. The treatment itself is very gentle and non-confrontational, and it is not known to provoke deterioration or side effects. The assigned therapist is available by phone between sessions in case any unexpected discomfort, fear, or pain arises. However, it should be emphasized that psychotherapy is not known to cause any of the aforementioned symptoms.

### Statistical analyses

As this is a pilot study with a new form of treatment that has not previously been used in Child and Adolescent Psychiatry, it has not been possible to make power calculations in relation to the number of participating patients. Instead, we have chosen a pragmatic approach and chosen the number of participants based on CFT studies with adults with schizophrenia. This is also in accordance with the recommendation that a pilot study of high confidence should have at least *n* = 50 participants (31).

Baseline data will be presented by descriptive statistics. Differences between young patients with voice hearing and patients with FES voice hearing will be analyzed by logistic regression analyses (continuous data) and the chi-square test (categorical data).

Generalized linear models can be used to examine differences, relative changes, or ratios between different time points. Effect sizes will be related to differences in mean change. This model allows for the inclusion of confounders or relevant covariates such as gender.

## Discussion

The treatment manual has been written based on clinical work with young voice hearers aged 8–20 years in a psychiatric hospital setting. Before writing the treatment manual, an extensive literature search was made, without identifying similar studies or approaches targeting this age group. The manual has been tested in its current form on young clinical patients for session time estimates and language appropriateness. From previous clinical experience, we know that young voice hearers are attending the psychiatric hospitals of Southern Denmark. We cannot, however, be certain whether they will accept being part of a research study, and there remains some uncertainty as to whether sufficient participants can be recruited. Should recruitment prove insufficient, we will contact services in the municipality that are in contact with a larger number of children and teenagers. The study is also dependent on recruiting professionals who are willing to deliver the treatment. As an incentive, we are planning to offer frequent supervision and a thorough 2-day introduction to the principles of CFT and the manual.

A defining feature of the ECHO study is the active involvement of primary caregivers in half of all sessions. Family involvement in early psychosis is supported by a strong evidence base. Claxton et al. (32) found in a systematic review and meta-analysis that family interventions significantly reduced psychotic symptoms and shifted caregivers from high to low expressed emotion, with benefits extending to caregiver wellbeing. The goal is not to position caregivers as co-therapists, but to build a shared language around voice hearing and mental states. This is grounded in attachment theory, which holds that secure interpersonal relationships buffer against psychiatric distress and support recovery (33), a proposition supported by van Bussel et al (34) meta-analysis linking insecure attachment to poorer outcomes across symptomatic, social, and personal recovery domains in psychotic disorders. This is also in line with the theoretical thinking in CFT.

Extending this relational focus, the audio recording intervention—in which caregivers listen to a recording approximating the content of the young person’s voices—aims to close the experiential gap between the young person and those closest to them. Research shows that voice content is meaningfully linked to personal history and interpersonal context, and that voices can be understood as dissociated components of the self arising from trauma or interpersonal stress (35). Yet, clinicians and parents often lack the confidence to engage directly with this content (15). By giving concrete form to an otherwise invisible experience, the recording may reduce isolation and provide an opening to more dialogue within the family.

The clinical comparison group of young adults with FES serves an exploratory purpose: to examine whether a subgroup of adolescent voice hearers resembles patients with FES in social cognition, voluntary voice control, and social connectedness. Trajectory modeling in the OPUS cohort has shown that subgroups of patients with FES follow markedly different long-term courses of AHs (36), underscoring the value of early subgroup identification for preventive intervention. It should, however, be noted that these analyses are hypothesis-generating rather than confirmatory.

The mirror-image design controls for time-invariant individual characteristics but carries limitations. Effect sizes should therefore be interpreted conservatively. The primary aims of this pilot are to establish feasibility and acceptability, and to deepen understanding of young voice hearers as a clinical population—forming the foundation for a future randomized controlled trial.

### Limitations

The study is limited by its design. We aim to study and treat a phenomenon, which typically in psychiatry is regarded as a symptom. Treatment as usual does not focus on voice hearing but almost considers it a by-product of psychiatric illness. We have chosen a mirror design to compensate for this issue, knowing that a two-armed design would be stronger. The main focus is to access the efficacy and the feasibility in terms of whether it is acceptable to the participants and obtain more knowledge about young voice hearers.

### Impact

If we find the desired effect of the new CFT treatment, the treatment can relatively easily be spread more widely in Child and Adolescent Psychiatry, municipal or school treatment programs as voice hearing is a transdiagnostic phenomenon, and the current treatment manual is clear and realistic to implement in daily clinical practice.

The project is in accordance with the UN’s Sustainable Development Goals with a particular focus on Goal 3: Ensure healthy lives and promote wellbeing for all and at all ages.
